# Massively parallel chaos for LiDAR

**DOI:** 10.1038/s41377-023-01136-z

**Published:** 2023-04-18

**Authors:** Shaohua Yu

**Affiliations:** Peng Cheng Laboratory, Shenzhen, 518055 China

**Keywords:** Frequency combs, Nonlinear optics

*Nature Photonics* **17**, 306–314 (2023)

10.1038/s41566-023-01158-4

LiDAR is an essential sensor for Level 3+ autonomous driving due to its unparalleled ability of accurate 3D mapping in real-time. However, the congestion problem, which occurs when their channel occupations overlap in time or frequency domain, has prevented the allowed channel number of individual LiDAR but also limits the density of working devices. This challenge, combined with the significantly increased cost when building the parallel LiDAR systems, has been stalling the progression of LiDAR technologies. A team of researchers from Peking University and the University of California, Santa Barbara, has found a promising new solution by using chaotic comb. This massively parallel chaotic source has inherent self-chaotic properties, that make each channel orthogonal to each other, showing good immunity to other LiDAR signals and single PD detection capability. This advancement has the potential to reshape the LiDAR ecosystem, providing a low-cost, efficient, and reliable solution for the next generation of LiDAR technologies. The simplicity of its architecture combined with its exceptional performance opens up a new era of LiDAR technology, with endless possibilities for further development and application.

